# Supplementary material for: Clinical and Behavioural Heterogeneity Among Women at Increased Risk for Gestational Diabetes: A Four-Country Analysis
Source: Int J Environ Res Public Health. 2025 Jun 27;22(7):1022. doi: 10.3390/ijerph22071022 (PMC12294607; doi:10.3390/ijerph22071022)
Supplement: Supplementary file 1 [file ijerph-22-01022-s001.zip › ijerph-3644644-supplementary.pdf]

Supplementary table S1. Normality and homogeneity of variance statistics for continuous variables

	**Kolmogorov-Smirnov Test**			**Levene's Test (based on mean)**		
	Statistic	df	P	Levene Statistic	df	P
Gestation (weeks) (n=798)	0.301	537	<0.001	24.839	3	<0.001
Age, mean (SD) (n=804)	0.139	706	<0.001	2.090	3	0.100
BMI (kg/m^2, mean (SD) (n=804)	0.121	706	<0.001	10.151	3	<0.001
BP Systolic, mean (SD) (n=631)	0.061	537	<0.001	2.499	3	0.059
BP Diastolic, mean (SD) (n=631)	0.085	537	<0.001	4.592	3	0.003
Dietary intake (n= 483)						
Energy (kcal/day)	0.073	483	<0.001	0.367	3	0.777
Fat (g/day)	0.070	483	<0.001	2.547	3	0.055
Protein (g/day)	0.064	483	<0.001	0.343	3	0.795
Carbohydrate (g/day)	0.095	483	<0.001	3.992	3	0.008
Activity (METs/week, n= 716)						
Total	0.109	706	<0.001	1.962	3	0.118
Household activities	0.105	706	<0.001	0.667	3	0.573
Occupational activities	0.198	706	<0.001	4.531	3	0.004
Sport/exercise activities	0.149	706	<0.001	3.409	3	0.017
Transportation activities	0.156	706	<0.001	0.650	3	0.583
Sedentary activities	0.046	706	0.001	1.492	3	0.215
EQ5D visual analogue scale (n= 732)	0.165	706	<0.001	0.772	3	0.510
